# ALCOHOL CONSUMPTION AND DECREASED SERUM Α-KLOTHO, AN ANTI-AGING HORMONE, LEVELS AMONG US ADULT PARTICIPANTS

**DOI:** 10.1093/geroni/igad104.3175

**Published:** 2023-12-21

**Authors:** Dylan Fromm, Jianmin Zhu, Wonjun Kim, Yudan Wei

**Affiliations:** Mercer University School of Medicine, Macon, Georgia, United States; Fort Valley State University, Fort Valley, Georgia, United States; Mercer University School of Medicine, Macon, Georgia, United States; Mercer University School of Medicine, Macon, Georgia, United States

## Abstract

**Background:**

The Klotho protein is widely accepted to be a prominent anti-aging biomarker for health and longevity. The relationship between alcohol intake and serum levels of soluble ɑ-Klotho is not well understood.

**Methods:**

In this study, we explored this relationship in a nationally representative sample of US adults. A total of 3970 male and female adults aged 40-79 years from the 2013-2016 National Health and Nutrition Examination Surveys were analyzed using multiple general linear models. Participants were classified as non-drinkers, light drinkers (consumed on average < 4 drinks in males and < 3 drinks in females per day), and heavy drinkers (consumed on average ≥4 drinks in males and ≥3 drinks in females per day), according to a national guideline.

**Results:**

Significantly decreased serum ɑ-Klotho levels were found in both light drinkers and heavy drinkers, with the weighted geometric mean of 785.18 pg/mL (p = 0.0007) in light drinkers and 750.96 pg/mL (p = <.0001) in heavy drinkers, compared to that in non-drinkers (827.19 pg/mL). After adjusting for covariates, light drinking was associated with a 3.66% (p = 0.0081) decrease in serum ɑ-Klotho levels, and heavy drinking with an 8.07% decrease (p = <.0001) in ɑ-Klotho levels, as compared with non-alcohol drinking. Similarly, binge drinking was also associated in a 5.22% (p = <.0001) decrease in serum ɑ-Klotho, compared to non-binge drinking.

**Conclusion:**

This study demonstrates a significantly inverse relationship between alcohol consumption and serum ɑ-Klotho levels in middle-aged and older adults. Prospective studies are warranted to elucidate these interactions.

